# PIWI proteins contribute to apoptosis during the UPR in human airway epithelial cells

**DOI:** 10.1038/s41598-018-34861-2

**Published:** 2018-11-06

**Authors:** Magdalena Gebert, Sylwia Bartoszewska, Anna Janaszak-Jasiecka, Adrianna Moszyńska, Aleksandra Cabaj, Jarosław Króliczewski, Piotr Madanecki, Renata J. Ochocka, David K. Crossman, James F. Collawn, Rafal Bartoszewski

**Affiliations:** 1 Department of Biology and Pharmaceutical Botany, Medical University of Gdansk, Gdansk, Poland; 2 Department of Inorganic Chemistry, Medical University of Gdansk, Gdansk, Poland; 3 Laboratory of Bioinformatics, Nencki Institute of Experimental Biology of the Polish Academy of Sciences, Warsaw, Poland; 4 Department of Genetics, Heflin Center for Genomic Science, University of Alabama at Birmingham, Birmingham, USA; 5 Department of Cell, Developmental and Integrative Biology, University of Alabama at Birmingham, Birmingham, USA

## Abstract

Small noncoding microRNAs (miRNAs) post-transcriptionally regulate a large portion of the human transcriptome. miRNAs have been shown to play an important role in the unfolded protein response (UPR), a cellular adaptive mechanism that is important in alleviating endoplasmic reticulum (ER) stress and promoting cell recovery. Another class of small noncoding RNAs, the Piwi-interacting RNAs (piRNAs) together with PIWI proteins, was originally shown to play a role as repressors of germline transposable elements. More recent studies, however, indicate that P-element induced WImpy proteins (PIWI proteins) and piRNAs also regulate mRNA levels in somatic tissues. Using genome-wide small RNA next generation sequencing, cell viability assays, and caspase activity assays in human airway epithelial cells, we demonstrate that ER stress specifically up-regulates total piRNA expression profiles, and these changes correlate with UPR-induced apoptosis as shown by up-regulation of two pro-apoptotic factor mRNAs, *CHOP* and *NOXA*. Furthermore, siRNA knockdown of *PIWIL2* and *PIWIL4*, two proteins involved in piRNA function, attenuates UPR-related cell death, inhibits piRNA expression, and inhibits the up-regulation of *CHOP* and *NOXA* mRNA expression. Hence, we provide evidence that PIWIL2 and PIWIL4 proteins, and potentially the up-regulated piRNAs, constitute a novel epigenetic mechanism that control cellular fate during the UPR.

## Introduction

The endoplasmic reticulum (ER) is the central organelle governing the synthesis, folding and post-translational modifications of membrane and secretory proteins. The disruption of ER homeostasis, termed as ER stress, can occur in a number of ways through protein overload, excessive protein misfolding, post-translational modifications or by changing the ion milieu inside the ER. The ER stress activates the unfolded protein response (UPR), a multifunctional signaling pathway with distinct sensors and targets that regulate gene expression^1^. The UPR serves primarily as a cellular adaptive mechanism that alleviates ER stress by activating multiple cellular pathways to restore ER homeostasis. If this cellular stress persists, however, or when the recovery mechanisms are inefficient, activation of the apoptotic cascades lead to cell death^2^. UPR-associated cell death contributes to the pathomechanisms of numerous human diseases including diabetes mellitus^3^, neurodegenerative disorders^4^, certain types of cancer, chronic inflammation, and certain forms of protein conformational diseases that are characterized by the decreased ability of cells to respond to stress^5^. In contrast, exaggerated stress responses in epithelial cells that are most likely to suffer from environmental stressors may also lead to complex pathological symptoms or cancer^6^. Therefore, it is critically important to understand the mechanisms regulating cell fate during UPR in order to develop novel interventions for treating these disorders.

Recently, a group of small non-coding RNAs (ncRNAs), microRNAs (miRNAs), has been shown to play a role in UPR^7^. However, the potential role of other classes of small ncRNAs in UPR signaling is relatively unknown. Here, we show that PIWI proteins, which associate with a novel class of small RNAs known as piwi-interacting RNAs (piRNAs), may play a role during UPR in determining cell fate. piRNAs are small, ∼23–30-nucleotide, endogenous RNAs that are generated in a Dicer-independent mechanism from long single stranded precursors^8^. Although the initial studies assigned piRNAs as repressors of mammalian germline transposable elements (TE), emerging evidence suggests that they may have other functions that affect mRNA levels and may provide another mechanism for regulating cellular events^8–10^. The piRNA-dependent mechanism of mRNA decay has been identified in *Drosophila* embryos^11,12^. TE silencing by piRNAs has been shown to be similar to that of other RNA-based silencing events in that piRNAs bound to PIWI proteins target mRNAs through RNA base pairing and the mRNAs are then cleaved by the endonuclease activity of the PIWI proteins (reviewed in^13^). More importantly, recent reports of mitochondrial DNA-encoded piRNAs and PIWI proteins in mammalian somatic cell lines indicated a role in cellular stress responses^14^. In conjunction with this, the role of piRNAs and PIWI proteins in cancer has been extensively studied^15,16^. Consistent with emerging roles for piRNAs in somatic cells, piRNA-like-163’s (piR-L-163) biological function was reported in human bronchial epithelial cells (HBECs)^17^. Furthermore, the human airway epithelia represent the first line of defense against invading pathogens and environmental stressors in the lung. However, often the exposure of airway epithelia to these stress factors disturbs ER homeostasis (ER stress) and leads to activation of the unfolded protein response (UPR) pathway. Despite the number of reports that piRNA expression has been observed in human somatic cells such as cancer cells^18^, the extent of PIWI protein and piRNA expression, and more importantly, their functional roles in mammalian somatic tissues remains fairly limited.

The studies presented herein show that ER stress specifically affects piRNA sequence expression profiles, and siRNA knockdown of PIWI proteins blocks UPR-induced cell death and interestingly decreases total piRNA expression. These results indicate that PIWI proteins, perhaps through a piRNA-directed pathway, constitute a novel epigenetic mechanism that regulates the cellular fate of human airway epithelial cells during the UPR.

## Material and Methods

### Cell lines and culture conditions

16HBE14o- cells were obtained as previously described^19,20^. HeLa cells were obtained from ATCC. Cells were cultured in Minimum Essential Modified Eagle’s Medium (Invitrogen) with 10% fetal bovine serum in a humidified incubator at 37 °C in 5% CO_2_ in 6-well plates and allowed to grow to 70–80% confluence prior to the start of the experiments.

### Induction of ER stress and activation of the UPR

Pharmacological induction of ER stress and activation of the UPR was performed according to previously described methods^21,22^. Briefly, cells were treated with ALLN (calpain inhibitor I; 100 μM, Sigma-Aldrich, A6185) or tunicamycin (2.5 μg/ml, Sigma-Aldrich, T7765) for the time periods specified.

### Isolation of RNA and small ncRNA

Total RNA containing the small ncRNA fraction was isolated using miRNeasy kit (Qiagen, 217004). RNA concentrations were calculated based on the absorbance at 260 nm. RNA samples were stored at −70 °C until use.

### Next generation small RNAs sequencing analyses

16HBE14o- cells were used for the RNA isolation and analyses. Small RNA sequencing libraries were prepared using QIAseq miRNA library kit (Qiagen) following the manufacturer’s instructions and followed by sequencing on an Illumina NextSeq instrument. Using Qiagen’s Gene Globe Software, sequencing reads were aligned to the human reference genome assembly (hg19) followed by transcript assembly and estimation of the relative abundances. The analysis of the differential expression of small RNAs between control and experimental samples were performed with geNorm^23^ in the Gene Globe Software.

### Measurement of mRNA levels using quantitative Real Time PCR (qRT-PCR)

We used TaqManOne-Step RT-PCR Master MixReagents (Applied Biosystems) as described previously^21,22,24^ using the manufacturer’s protocol. The relative expressions were calculated using the comparative relative standard curve method^25^. We used TATA-binding protein (*TBP*) *mRNA* as the relative control for our studies. TaqMan probes ids used were: *TBP* - Hs4332659_m1; *BIP* - Hs00607129_gH; *CHOP* - Hs00358796_g1; *PIWIL2* - Hs01032720_m1; *PIWIL4* – Hs00381509_m1;; *NOXA* Hs00560402_m1.

### siRNA transfections

siRNA against *PIWIL2* (Ambion assay id s30226) and *PIWIL4* (Ambion assay id s44573) were purchased from Ambion. 16HBE14o- cells were transfected/co-transfected using the Lipofectamine RNAiMax (Invitrogen, 13778030) according to manufacturer’s protocol. The siRNAs were used at final concentrations of 40 nM. The transfected cells were cultured for 2 days prior to further analysis. The degree of PIWIL2 and PIWIL4 knockdowns was determined by Western blot. Ambion siRNA Negative Control 1 (Ambion assay id MC22484) was used as a control.

### Western Blots

Cells were lysed in RIPA buffer (150 mM NaCl, 1% NP-40, 0.5% sodium deoxycholate, 0.1% SDS, 50 mM Tris- HCl, pH 8.0) supplemented with protease Inhibitor Complete Mini (Roche, 000000011836170001) on ice for 15 min. The cell lysates were rotated at 4 °C for 30 min and the insoluble material was removed by centrifugation at 15,000 g for 15 min. Protein concentrations were determined by Bio-Rad Protein Assay using bovine serum albumin (BSA) as the standard. Following the normalization of protein concentrations, lysates were mixed with an equal volume of 2X Laemmli sample buffer and incubated for 5 min at 95 °C prior to separation by SDS PAGE on stain-free TGX gradient gels (Bio-Rad). Following SDS-PAGE, the proteins were transferred to polyvinylidene fluoride membranes (300 mA for 90 min at 4 °C). The membranes were then blocked with BSA (Sigma-Aldrich) dissolved in PBS/Tween-20 (3% BSA, 0.5% Tween-20 for 1–2 hours), followed by immunoblotting with the primary antibody specified for each experiment for PIWIL2 (Abcam, ab181340 diluted at 1:1000), PIWIL4 (Abcam ab 111714, diluted at 1:1000), and beta Actin (Abcam ab1801, diluted at 1:1000). After the washing steps, the membranes were incubated with goat anti-rabbit IgG (H + L) HRP-conjugated secondary antibodies (Bio-Rad) and detected using ECL (Amresco). Densitometry was performed using Image Lab software v. 4.1 (Bio-Rad).

### Cell viability assays

For real-time monitoring of cell viability, we used the Roche xCeligence system as described previously in^24^. Briefly, 16HBEo- cells (2000 cells per well) were seeded in the 16 well PC plates 24 h prior to treatment. Control cells were cultured in a presence of DMSO vehicle. Treated cells were incubated with ER stressors for the next 24 h, and every 15 minutes the cell conductances (cell index) were recorded. All experiments were performed in triplicate with 2 independent repeats. Similar assays were performed for the *PIWIL2* and *PIWIL4* silencing. Briefly, the day after transfection with control siRNA, siRNA against *PIWIL2 or PIWIL4*, or co-transfection with both siRNAs against *PIWIL2* and *PIWIL4*, the cells were seeded in 16-well PC plates (ACEA, 00300600890) 24 h prior to treatment. Control cells were cultured in a presence of DMSO vehicle. Treated cells were incubated with ER stressors for the next 24 h, and every 15 minutes the cell conductances were recorded. RTCA software v. 1.2.1 (ACEA) was used to calculate the normalized cell index and the cells’ growth curve slopes.

#### Monitoring caspase 3 and caspase 7 activity

The caspase 7 is considered to be redundant with caspase 3 because these enzymes share an optimal peptide recognition sequence and have several endogenous protein substrates in common^26^. Furthermore, both of these enzymes are activated by the initiators caspase 8 and caspase 9^26^. While our main goal was to assess caspase 3 activity, the commercially available assays do not distinguish between these two cysteine proteases. Therefore, we used the caspase-Glo 3/7 assay (Promega) to determine relative caspase activity. Briefly, the day after transfection with the specified siRNA, cells were seeded onto 96-well luminescence assay white plates with clear bottoms (Corning Inc., 3903). Next day cells were treated with ER stressors or vehicle (1% DMSO) for indicated time points. Following treatment, cells were washed with PBS and the Caspase-Glo 3/7 assay (Promega) was performed in accordance with the manufacturer’s instructions, using GloMax-Multi + Detection System (Promega). The results were normalized by subtracting the values obtained from the vehicle treatments.

### ER stress response element analysis

ER stress response elements (ERSE) consist of a pair of motifs that bind ATF6/XBP1 and NFY transcription factors that are separated by 9 nucleotides. Another response element is ERSE II, which looks similar to ERSE, but the binding of ATF6/XBP1 and NFY are separated by only 1 nucleotide and the motif for binding NFY has the reverse complementary sequence. Their consensus sequences are CCAAT-N_9_-CCACG and ATTGG-N-CCACG, respectively^27^. We used a Nencki Genomics Database^28^ (v. 79_1) webservice in Taverna Workbench client^29^ in order to obtain genomic coordinates of the motifs for ATF6/XBP1 and NFY in the regulatory regions ±10 kb region around the transcription start sites (TSSs) of *PIWIL2* and *PIWIL4* genes. Using a simple script, we searched for pairs of those motifs separated from each other by 1 to 9 nucleotides.

### Statistical analysis

Results were expressed as means ± standard deviations (SD). Statistical significance among means was determined using the Student’s t-test (two samples, paired and unpaired) or the Kruskal-Wallis One Way Analysis of Variance on Ranks^30^ and Dunn’s Method^31^ (ANOVA on ranks). The correlation coefficients and p values were calculated according to Spearman Rank-Order Correlation^32^.

## Results

As our UPR cell fate model system, we used immortalized human bronchial epithelial cells (16HBE14o-) since these are commonly used for studying human airway physiology^19,20,33^. To determine when pharmacological ER stress induces UPR-related apoptosis, we first performed a time-course study and monitored 16HBE14o- cell responses after inducing UPR with the two classic UPR-inducing compounds, ALLN, a proteasome inhibitor, or tunicamycin, a glycosylation inhibitor^34,35^.

**Figure 1:**
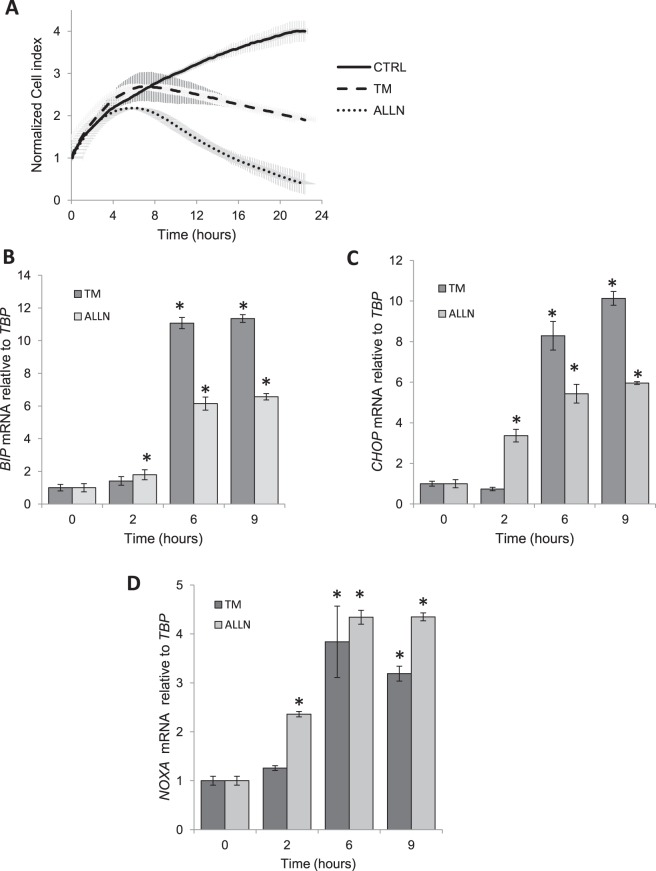
Real time analysis of 16HBE14o- cell survival during ER stress. (**A**) The cell conductances (expressed as normalized cell index) were accessed every 15 minutes following 24-hour treatment with TM (2.5 µg/ml), ALLN (100 µM) or DMSO (CTRL). The conductances were normalized to the last value prior to experiment start. The results from 3 measurements (n = 3) are plotted. ER stress-induced changes in *BIP* (**B**), *CHOP* (**C**), and *NOXA* (**D**) mRNA levels are shown. The results from 2 independent experiments (n = 8) are plotted normalized to *TBP* mRNA levels and expressed as a fold-change over the no stress controls. Error bars represent standard deviations. Significant changes (p < 0.05) are marked with an asterisk.

As shown in Fig. 1A using real time analysis of 16HBE14o- viability^24^, both stressors had no significant effect on the 16HBE14o- cell growth and survival analysis until approximately 4 hours. By 6 to 7 hours of treatment, however, the normalized cell index leveled off, and by 8 hours of treatment, the cell index numbers decreased, particularly in the ALLN-treated sample. Based on these observations, we propose that 2 hours represents the initial adaptive response to ER stress, 6 hours corresponds to cell fate decisions, and 9 hours represents the start of the UPR-related apoptosis in the 16HBE14o- cells.

To support this initial analysis, we isolated RNA samples in order to examine the mRNA levels of both pro-survival and pro-apoptotic UPR reporters. For the pro-survival reporter, we analyzed the 78-kDa glucose-regulated protein (*BIP*) mRNA levels^36,37^ and for two pro-apoptotic reporters, we analyzed the CCAAT-enhancer-binding protein homologous protein (*CHOP*)^38,39^ and *NOXA*^40^ mRNA levels. *BiP* mRNA expression was significantly induced by both stressors and reached maximal levels by 6 hours and remained elevated (Fig. 1B). The mRNA expression of *CHOP* and *NOXA* was significantly induced by ALLN at the 2 hour time point and reached maximum at 6 hours and remain elevated, whereas TM treatment resulted in significant *CHOP* and *NOXA* mRNA increases at 6 hours (Fig. 1C,D). Taken together, both the proteasome blockage and glycosylation inhibition induced UPR signaling at the 6 h time point, and this corresponded to the observed cell growth plateau seen in the cell index experiment (Fig. 1A).

**Figure 2:**
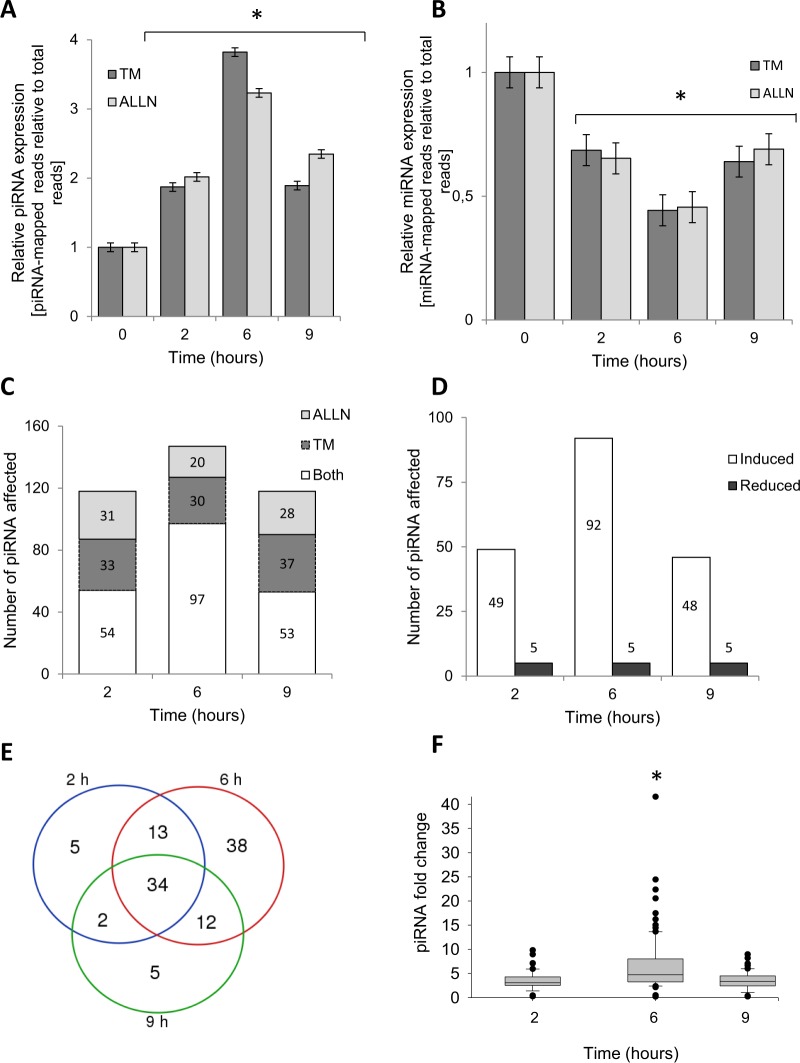
ER stress induces dynamic changes in piRNA and miRNA expression. (**A**) ER stress increases piRNA expression in 16HBE14o- cells. The mapped piRNA reads in NGS analysis were normalized to total mapped RNA reads and expressed as fold change over no stress control; TM (2.5 µg/ml), ALLN (100 µM). (**B**) ER stress reduces miRNA expression in 16HBE14o- cells. The mapped miRNA reads in NGS analysis were normalized to total mapped RNA reads and expressed as fold change over no stress control; TM (2.5 µg/ml), ALLN (100 µM). (**C**) Both ER stressors have a similar effect on piRNA expression. The number piRNAs significantly affected (two-fold relative to no stress control) are shown in the bar graph; ALLN (light grey), TM (dark grey), and both stressors (white). The number of piRNAs in each group is shown. (**D**) The ER stress distribution of piRNAs significantly increased (white) or decreased (grey) by ALLN and TM. (**E**) The Venn diagram of piRNAs affected by both ER stressors at 2, 6 and 9 hours. 34 piRNAs were continuously upregulated during the entire stress time course, another 34 of these RNAs were specifically induced at 6 hours only (38 specifically affected at this time point), whereas only 3 and 2 piRNAs each were induced during 2 and 9 hour time points (out of 5 specifically affected at each of this time points). (Supplemental Table 1) (**F**) Relative piRNA levels are significantly higher and maximal at the 6-hour time point after induction of ER stress. The piRNA levels (affected by both ER stressors) were expressed as fold change relative to no stress control, and the groups were compared with ANOVA on ranks. Significant changes (p < 0.05) are marked with an asterisk. The respective sequence length distribution profiles are provided in Supplemental Fig. 1.

Next, we obtained RNA samples under control conditions (no stress) as well as after 2, 6 and 9 hours of treatment with either ALLN or TM, and subjected them to genome-wide small RNA next generation sequencing followed by bioinformatic analysis. Our analysis of the ER stress-related relative global small RNA expression changes indicates that the piRNA expression profiles correlated well with *BIP* and *CHOP* mRNA induction profiles (Fig. 2A), whereas the global miRNA expression was reduced more than 50% at 6 hours following ER stress induction (Fig. 2B). These data were confirmed by the sequence length distribution profiles that the piRNAs were elevated during both ER stresses using FastQC software (Supplemental Fig. 1, Table 3). Given that the piRNAs were maximally elevated during ER stress, we focused our further analysis on these small ncRNAs.

Although in all time points analyzed, both stressors had very similar effects on relative piRNA expression (Fig. 2C, Supplemental Table 1), we only focused on piRNAs that were induced or reduced more than 2 fold by both ALLN and TM. Based on this, 97 unique piRNAs were induced at 6 hours, while only 12 were reduced during UPR (Supplemental Table 1, Fig. 2C,D). Although the 34 unique piRNAs were continuously up regulated during the entire stress time course, another 34 of these RNAs were specifically induced at 6 hours only (Supplemental Table 1, Fig. 2E). Interestingly, no piRNA was continuously reduced during entire time course (Supplemental Table 1). Furthermore, as shown in Fig. 2F, for the majority of the identified piRNAs, their relative levels were significantly higher at the 6 h time point and this represents what we call the cell fate decision stage.

**Figure 3:**
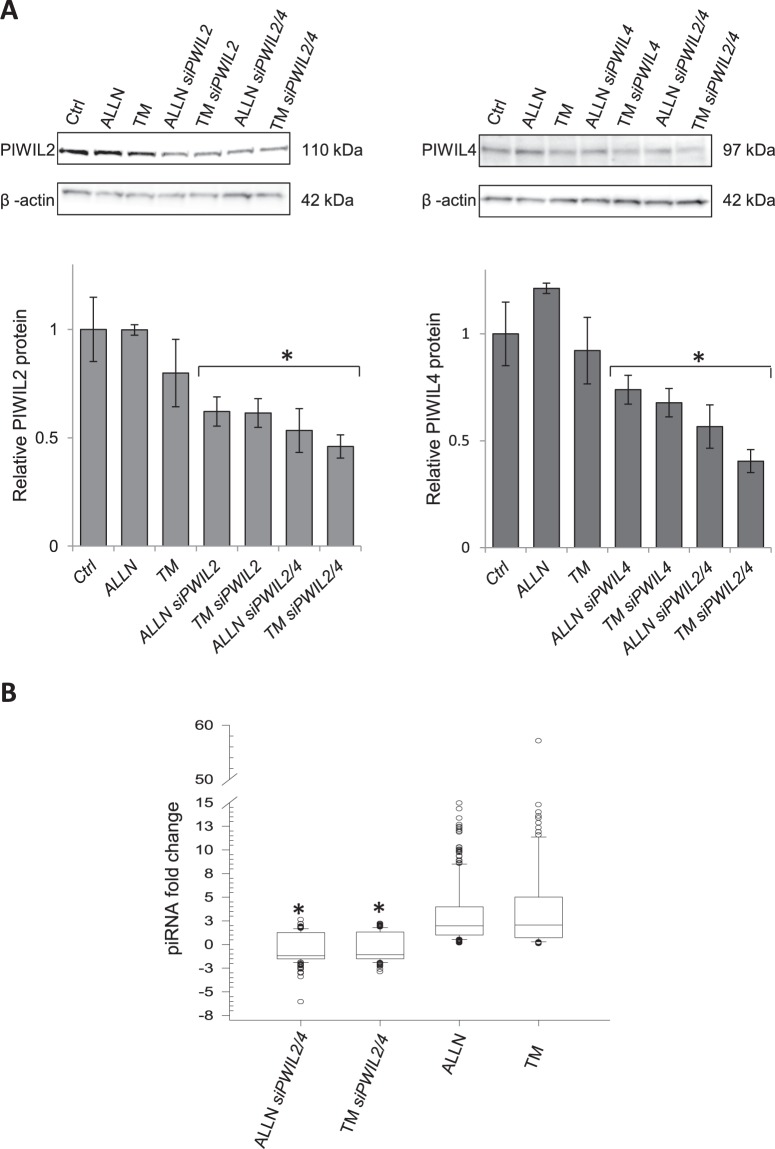
Silencing of *PIWIL2* and *PIWIL4* decreases piRNA expression during ER stress. (**A**) 16HBE14o- cells were transfected with siRNA against *PIWIL2* (siPIWIL2), *PIWIL4* (siPIWIL4) or co-transfected with both (siPIWL2/4) and treated with TM (2.5 µg/ml) or ALLN (100 µM) for 6 hours, and the corresponding changes in PIWIL2 and PIWIL4 protein levels were detected with SDS-PAGE and Western Blot and normalized to the β-Actin (2 µg of total protein per lane). The experiments were repeated twice and the error bars represent standard errors. Significant changes (p < 0.05) are marked with an asterisk. The original blots and gels are provided in Supplemental Fig. 4. (**B**) Reduction of *PIWIL2* and *PIWIL4* relative expression with siRNAs (siPIWIL2/4) lowers piRNAs levels after 6 hours of ER stress. The piRNAs levels were accessed by next generation small RNA sequencing and expressed as fold change relative to the no stress control. The groups (ALLN and ALLN siPIWIL2/4; TM and TM siPWIL2/4) were compared using ANOVA on ranks. Significant changes (p < 0.05) are marked with an asterisk.

In order to test if the observed changes in piRNA expression were ER stress-specific, we inhibited piRNA function by silencing PIWIL proteins^8,9^ and followed the effects on the global piRNA expression profile after 6 hours of ER stress (ALLN and TM). Although four isoforms of PIWIL proteins have been identified in the human genome^9^, the *PIWIL1* (*Hiwi*) and *PIWIL3* transcripts are not present in 16HBE14o- cells or in HeLa cells (Supplemental Fig. 2), and therefore we only used siRNAs against *PIWIL2* (*Hili*) and *PIWIL4* (*Hiwi2*). During ER stress, the silencing of each of these PIWIL proteins separately and with simultaneous co-silencing were effective and reduced these proteins levels by about 50% for both ER stressors (Fig. 3A). Hence, to ensure maximal effect on piRNA function, we analyzed co-silencing of both *PIWL2* and *PIWIL4* after 6 hours of ER stress. Briefly, RNA samples obtained after 6 hours of ALLN- or TM-induced ER stress with and without *PIWIL2* and *PIWIL4* silencing were subjected to genome-wide small RNA next generation sequencing followed by bioinformatic analysis. When *PIWIL2* and *PIWIL4* expression were reduced, the ER stress effects on piRNA expression were reduced, with the majority of piRNA either reduced or unaffected (Fig. 3B, Supplemental Table 2). Furthermore 6 piRNAs were significantly downregulated, and one (hsa_piR_019628) upregulated (Supplemental Table 2). Interestingly, this piRNA (hsa_piR_019628) was reduced by ER stress without silencing (Supplemental Table 2). The results indicate that that the ER stress-related changes in piRNA levels require the activity of PIWIL proteins.

**Figure 4:**
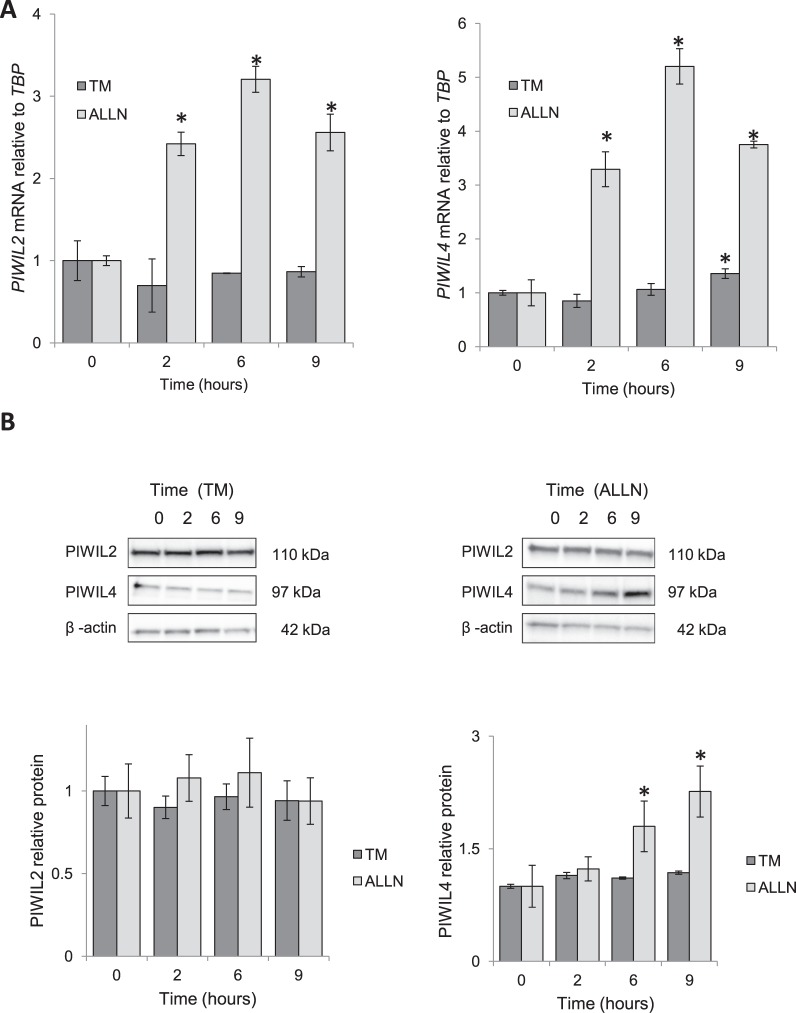
ER stressors have different effects on the expression of *PIWIL2 and PIWIL4* mRNA and protein. (**A**) 16HBE14o- cells were treated with TM (2.5 µg/ml) or ALLN (100 µM) for time points specified and *PIWIL2* and *PIWIL4* mRNA levels from 2 independent experiments (n = 8) are plotted normalized to *TBP* mRNA levels and expressed as a fold-change over the no stress controls. Error bars represent standard deviations. Significant changes (p < 0.05) are marked with an asterisk. (**B**) The corresponding changes of PIWIL2 and PIWIL4 protein levels were detected with SDS-PAGE and Western Blot and normalized to the β-Actin and 2 individual samples (2 µg of total protein per lane) were tested for each treatment and the experiments were repeated twice. The original blots and gels are provided in Supplemental Fig. 4.

In the next stage, we analyzed mRNA and protein expression of both of PIWIL2 and PIWIL4 during ER stress. Depending on ER stress induction mechanism, different effects on *PIWIL2* and *PIWIL4* mRNA levels were observed. As shown in Fig. 4A, the proteasome inhibition with ALLN significantly induced *PIWIL2* and *PIWIL4* mRNAs, whereas the glycosylation inhibitor treatment had minimal effect on these transcripts. Surprisingly, the corresponding PIWIL2 protein levels remained unaffected by both stressors, while the PIWIL4 protein levels were induced at 6 hours and 9 hours with ALLN treatment (Fig. 4B). Furthermore, with bioinformatic analysis we could not identify any ER stress response elements (ERSE and ERSE II) in the genomic sequences of *PIWIL2* and *PIWIL4* in the 10 kb regions around their transcription start sites (TSSs). The results indicate that while the mRNA levels of *PIWIL2* and *PIWIL4* increase during the ER stress in 16HBE14o-, only the PIWIL4 protein levels were elevated during ALLN treatment.

In human cancer cells, piRNAs and PIWI proteins have been proposed to prevent apoptotic signaling via increasing activity of pro-survival factors^9^. Hence, UPR increases in the piRNA levels could potentially prevent apoptosis and thus contribute to cell survival. To test this hypothesis, we followed 16HBE14o- cell survival during both types of ER stress along with a simultaneous impairment of piRNA biogenesis and function by co-silencing *PIWIL2* and *PIWIL4* or by silencing each of these proteins separately.

**Figure 5:**
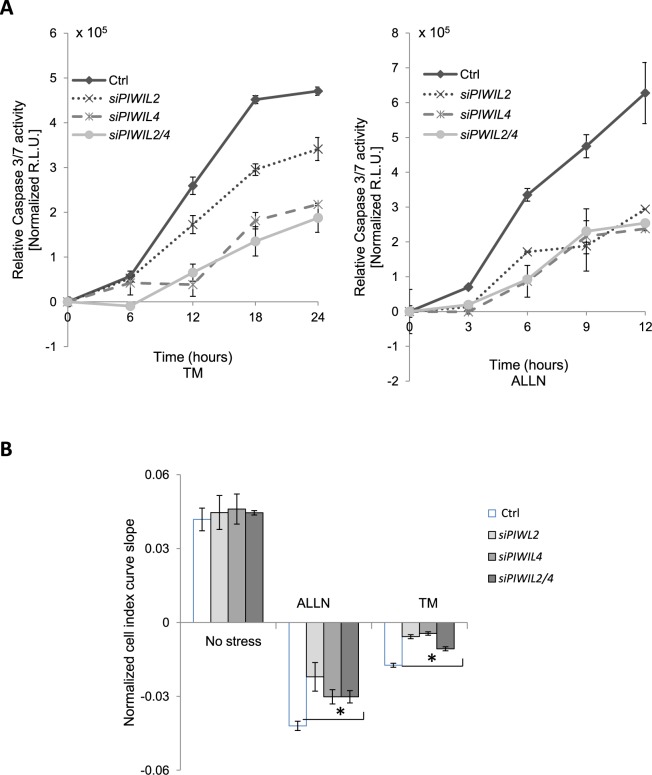
Silencing of *PIWIL2* and *PIWIL4* decreases caspase 3/7 activity. (**A**) 16HBE14o- cells were transfected with control siRNA (Ctrl); *PIWIL2* siRNA (siPWIL2); *PIWIL4* siRNA (siPWIL4); or both *PIWIL2* and *PIWIL4* siRNAs (siPWIL2/4), and treated with ER stressors (TM (2.5 µg/ml) - left panel, ALLN (100 µM) - right panel) for time points specified. The caspase 3/7 activity was monitored by luminescence and expressed in Relative Light Units (R.L.U). Cells for each time point were seeded in triplicate, and the experiments repeated twice. Error bars represent standard derivation. (**B**) Corresponding real time analysis of 16HBE14o- cell survival during ER stress during *PIWIL* mRNA silencing. The cell conductances (expressed as normalized cell index) were accessed every 15 minutes following 24 hours of treatment with TM (2.5 µg/ml), ALLN (100 µM) or DMSO (CTRL). The conductances were normalized to the last value prior to experimental start. The results from 3 measurements (n = 3) are plotted as slopes obtained from the cell growth curves. Error bars represent standard deviations. The experiments were repeated twice. Significant changes (p < 0.05) are marked with an asterisk.

Surprisingly, silencing both *PIWIL2* and *PIWIL4* during ER stress in 16HBE14o- reduced the relative levels of caspase 3/7 activity compared to the ER stress control (Fig. 5A). In the control sample, there was a significant increase in caspase 3/7 activity after 6 hours. siRNA reduction of *PIWIL2* or *PIWIL4* or both, however, resulted in decreases in stress-related induction of caspase activity when compared to control siRNA. Similar data were obtained independently by real time analysis of 16HBE14o- viability, where ER stress-related cell death as indicated by the normalized cell index was significantly decreased upon PIWIL2 and PIWIL4 proteins reduction (Fig. 5B).

**Figure 6:**
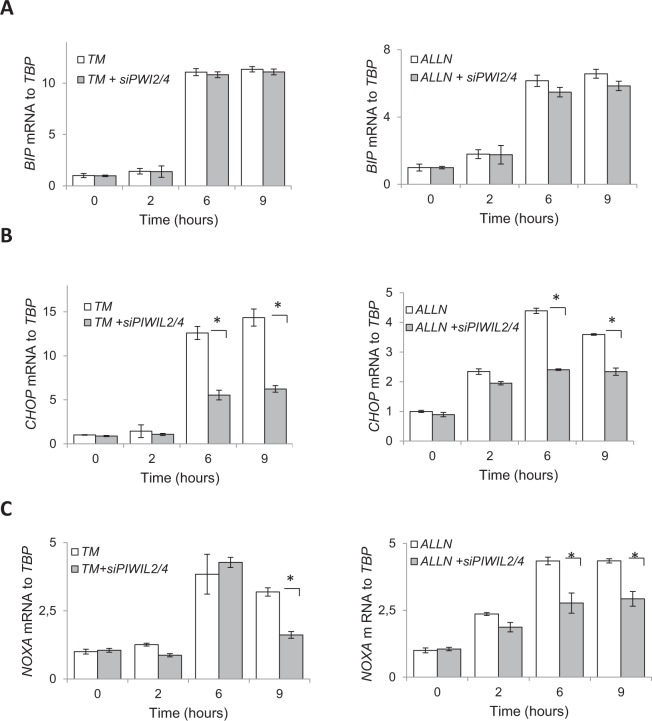
Silencing of *PIWIL2/4* decreases *CHOP* and *NOXA* mRNA expression levels during ER stress. 16HBE14o- cells were transfected with control siRNA or both *PIWIL2* and *PIWIL4* siRNAs (siPWIL2/4), and treated with ER stressors (TM (2.5 µg/ml) - left panels, ALLN (100 µM) - right panels) for time points specified. The corresponding mRNA levels of (**A**) *BIP*, (**B**) *CHOP*, and (**C**) *NOXA* from 2 independent experiments (n = 8) are plotted normalized to *TBP* mRNA levels and expressed as a fold-change over the no stress siRNA controls. Error bars represent standard deviations. Significant changes (p < 0.05) are marked with an asterisk.

The results suggest that ER stress induction of PIWI protein levels contributes to the pro-apoptotic arm of UPR. Next, we tested the consequences of impaired PIWI function on the expression levels of BIP and of known pro-apoptotic regulators of UPR. As shown in Fig. 6A,B, reduction of *PIWIL2* and *PIWIL4* expression during ER stress had no significant effect on BIP mRNA induction, but it did result in significantly lower levels of the pro-apoptotic *CHOP* transcript with both ER stressors. Similarly, *NOXA* expression was induced during ER stress, while siRNA knockdown of *PIWIL2/4* significantly reduced *NOXA* mRNA levels at 9 hours in the TM treated cells and at 6 and 9 hours in the ALLN treated cells (Fig. 6C). Taken together, these data indicate that *CHOP* and *NOXA* mRNA levels are enhanced during the ER stress response by the expression of the PIWI2/4 proteins, and that these PIWI proteins are novel components of the ER stress-induced apoptotic pathway and contribute to ER stress-induced apoptosis.

## Discussion

It is well established that unmitigated ER stress results in cellular functions deterioration, often leading to cell death^41^. To date, a number of mechanisms have been identified to be involved in ER stress programmed cell death including the induction of the pro-apoptotic transcription factor CHOP^42^, activation of pro-apoptotic cJUN NH_2_-terminal kinase (JNK) pathway^43^, proteolytic cleavage of ER-associated caspase-4 in human^44^ as well as mitochondria-initiated apoptosis dependence on p53 that involves the pro-apoptotic PUMA and NOXA proteins^40^. Despite these important discoveries, the majority of research has been performed in cancer cell lines. Recently, a class of small non-coding RNAs, miRNA, has been linked to the unfolded protein response pathway, and shown that these miRNAs through their posttranscriptional effects on multiple components of UPR can determine cell fate during ER stress^45,46^ (reviewed in^7,47,48^). Although this miRNA’s role in UPR remains extensively studied, the functions of other small non-coding RNA classes in this pathway remain relatively unknown^46^.

As proof-of-principle, we used a non-cancer but immortalized lung epithelial cell line (16HBE14o-) and utilized label-free, real time monitoring of cell survival during ER stress to determine when the cell fate decision occurs. In these studies, ER stress-related cell growth inhibition occurs at about 6 hours post-induction and is accompanied by a significant induction of pro-apoptotic factors including *CHOP* and *NOXA* as well as induction of the main pro-survival factor *BIP*.

Although our studies were similar to a previous report on Jurkat pre-T- cells that did not examine piRNAs^46^, we found a decrease in the relative quantity of miRNA-mapped reads and also noted a large relative increase in piRNA-mapped reads (3–4 fold) that were most significantly induced during the cell fate decision stage (6 hours). Furthermore, different mechanisms of ER stress induction had very similar effects on piRNA expression profiles, and resulted mainly in induction of these RNAs, whereas the total miRNAs were reduced. Therefore, our data reveal the first example of piRNA up-regulation during ER stress induction.

Although piRNAs have been assigned a role in defense mechanism that protects animal germ cells from deleterious effects of transposons and maintains chromatin structure^8^, there is emerging evidence of their role in somatic cells that include regulation of cell proliferation, differentiation and survival (reviewed in^9^). Because one of the functions of mammalian piRNA is posttranscriptional repression of gene expression through promoting mRNA decay^8^, these RNAs could be an important factor in the pro-adaptive/pro-apoptotic molecular switch emanating from the ER. Indeed, earlier studies suggested that PIWIL proteins are responsible for piRNA maturation and function, promote cancer cells survival, and may rescue cells from various cellular stress insults, and may have effects independent of piRNAs^9,49–51^.

It is possible that ER stress-related changes in piRNA expression result from specific modulation of PIWIL protein activity. Indeed, recent studies show that during piRNA biogenesis, loading piRNA precursors into PIWIL proteins requires activity of heat shock protein 90 (Hsp90)^52^. Importantly, Hsp90 is also important regulator of UPR signaling^53^. Our studies also demonstrate that PIWIL2 and PIWIL4 protein expression are required to induce the expression of piRNAs during ER stress. Our studies, however, are correlative with regard to the role of piRNAs in this process and therefore the identification of the piRNA targets as well as their importance awaits further study. That being said, such experimental approaches with piRNAs are limited by the lack of verified piRNAs analogs and lack of tools that allow for piRNA isolation that are free of other small ncRNAs fractions.

In comparing our identified piRNA sequences with the piRNA sequences identified in a 2015 study in human lung bronchial epithelial cells (HBEs)^17^, we find that about 30% of piRNA sequences were identical in both studies (Supplemental Fig. 2)^17^. Furthermore, using the same methodical approach, we confirmed the presence of these piRNA sequences in HeLa cells (Supplemental Fig. 2) and observed that in both 16HBEs and HeLa cells, the piRNA expression profiles are closely correlated.

Although we observed total miRNA level reductions during ER stress, these RNAs are important modulators of UPR responses^7^, and previous studies reported their impact on *CHOP*^54^ and *NOXA* mRNA levels^55^. However, as shown in Supplemental Fig. 3, impairing miRNA function during UPR (via *DICER1* silencing) affects both the adaptive and apoptotic arms of UPR, whereas the effects of the *PIWIL2/4* genes silencing were limited to the apoptotic pathway only. Furthermore, we have identified miRNAs that could directly contribute to *CHOP* and *NOXA* upregulation during UPR (Supplemental Table 4), but their functional roles will require further validation.

In summary, we identified that ER stress induces piRNA expression and that PIWIL2/4 expression is required for apoptosis in human airway epithelial cells. Although further studies will be necessary to test the mechanism underlying PIWIL protein and potentially piRNA activity during ER stress conditions, the studies presented identify a novel relationship between PIWI proteins and the apoptotic arm of UPR in deciding cell fate during ER stress.

## Electronic supplementary material

Supplementary Info

Supplemental Table 1

Supplemental Table 2

## Data Availability

Deep sequencing data were deposited in Gene Expression Omnibus (GEO) at accession number: GSE117629.
