# Supplementary material for: PIWI proteins contribute to apoptosis during the UPR in human airway epithelial cells
Source: Sci Rep. 2018 Nov 6;8:16431. doi: 10.1038/s41598-018-34861-2 (PMC6219583; doi:10.1038/s41598-018-34861-2)
Supplement: Supplementary file 1 — Supplementary Info [file 41598_2018_34861_MOESM1_ESM.pdf]

Supplementary Info

PIWI proteins contribute to apoptosis during the UPR in human airway epithelial cells

Magdalena Gebert[1], Sylwia Bartoszewska[2¥], Anna Janaszak-Jasiecka[1¥], Adrianna Moszyńska[1], Aleksandra Cabaj[3], Jarosław Króliczewski[1], Piotr Madanecki[1], Renata J.Ochocka[1], David K. Crossman[4], James F. Collawn[5], Rafal Bartoszewski[1*]

Affiliations

[1]Department of Biology and Pharmaceutical Botany, Medical University of Gdansk, Gdansk, Poland

[2]Department of Inorganic Chemistry, Medical University of Gdansk, Gdansk, Poland

[3] Laboratory of Bioinformatics, Nencki Institute of Experimental Biology of the Polish Academy of Sciences, Warsaw, Poland

[4] Department of Genetics, Heflin Center for Genomic Science, University of Alabama at Birmingham, Birmingham, USA

[5]Department of Cell, Developmental and Integrative Biology, University of Alabama at Birmingham, Birmingham, USA

[¥] These authors contributed equally

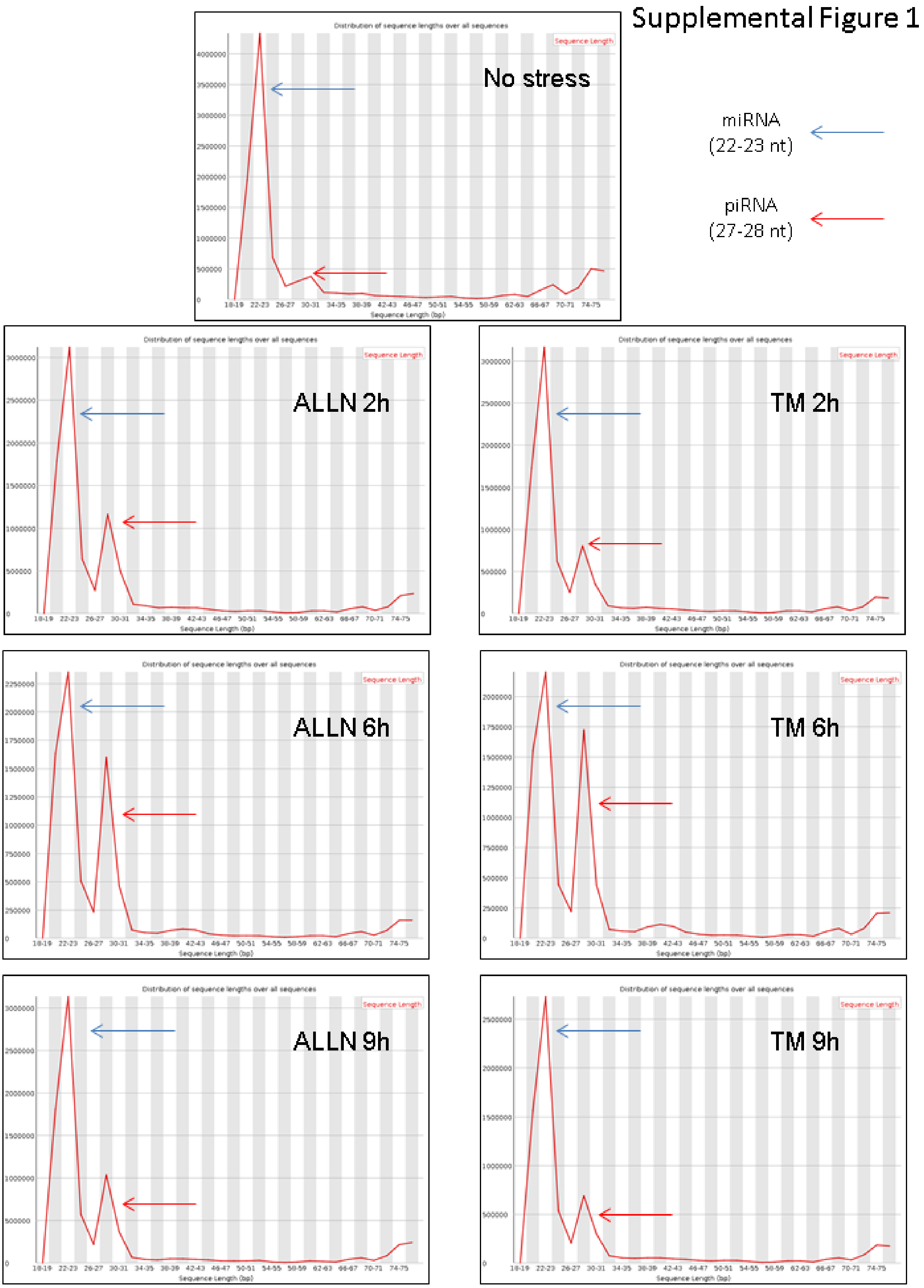

Supplemental Figure 1. The sequence length distribution profiles of small RNA sequencing during ER stress time course. The profiles were generated with use of FastQC software (FastQC A Quality Control tool for High Throughput Sequence Data *http://www.bioinformatics.babraham.ac.uk/projects/fastqc/* by S. Andrews).

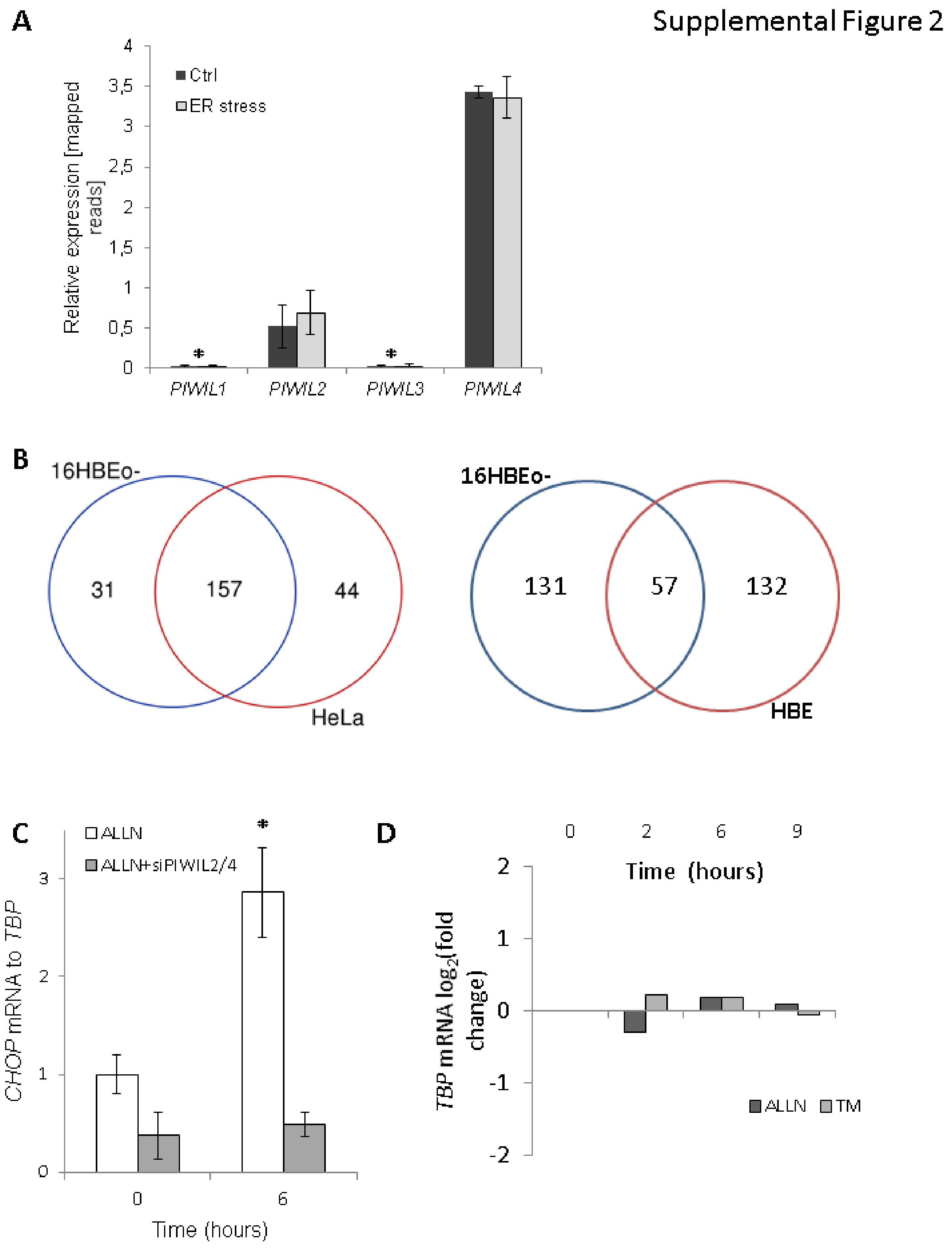

Supplemental Figure 2. (**A**) The NGS assessment of human *PIWIL1-4* gene expression in 16HBE14o- cells. The graph represents the average number of mapped reads (n6) for each of the genes. The Ctrl represents the expression in cells without ER stress, while ER stress

represents the expression in ALLN and TM treated 16HBE14o- cells. (**B**) The Venn diagram of piRNA sequences detected with NGS in 16HBEo-, HeLa and HBE cells [17]. (**C**) Silencing of *PIWIL2/4* decreases *CHOP* mRNA expression levels during ER stress. HeLa cells were transfected with control siRNA or both *PIWIL2* and *PIWIL4* siRNAs (si*PIWIL2/4*), and treated with the ER stressor (ALLN (100μM)) for 6 hours. The corresponding mRNA levels of CHOP (n=4) are plotted normalized to *TBP* mRNA levels and expressed as a fold-change over the no stress siRNA control. Error bars represent standard deviations. Significant changes ($p<0.05$) are marked with an asterisk. (**D**) *TBP* mRNA levels are not significantly affected during ER stress. *TBP* mRNA relative levels were obtained from corresponding RNASeq of ER stress exposed 16HBE o- cells and plotted as relative $\log_2$ fold change.

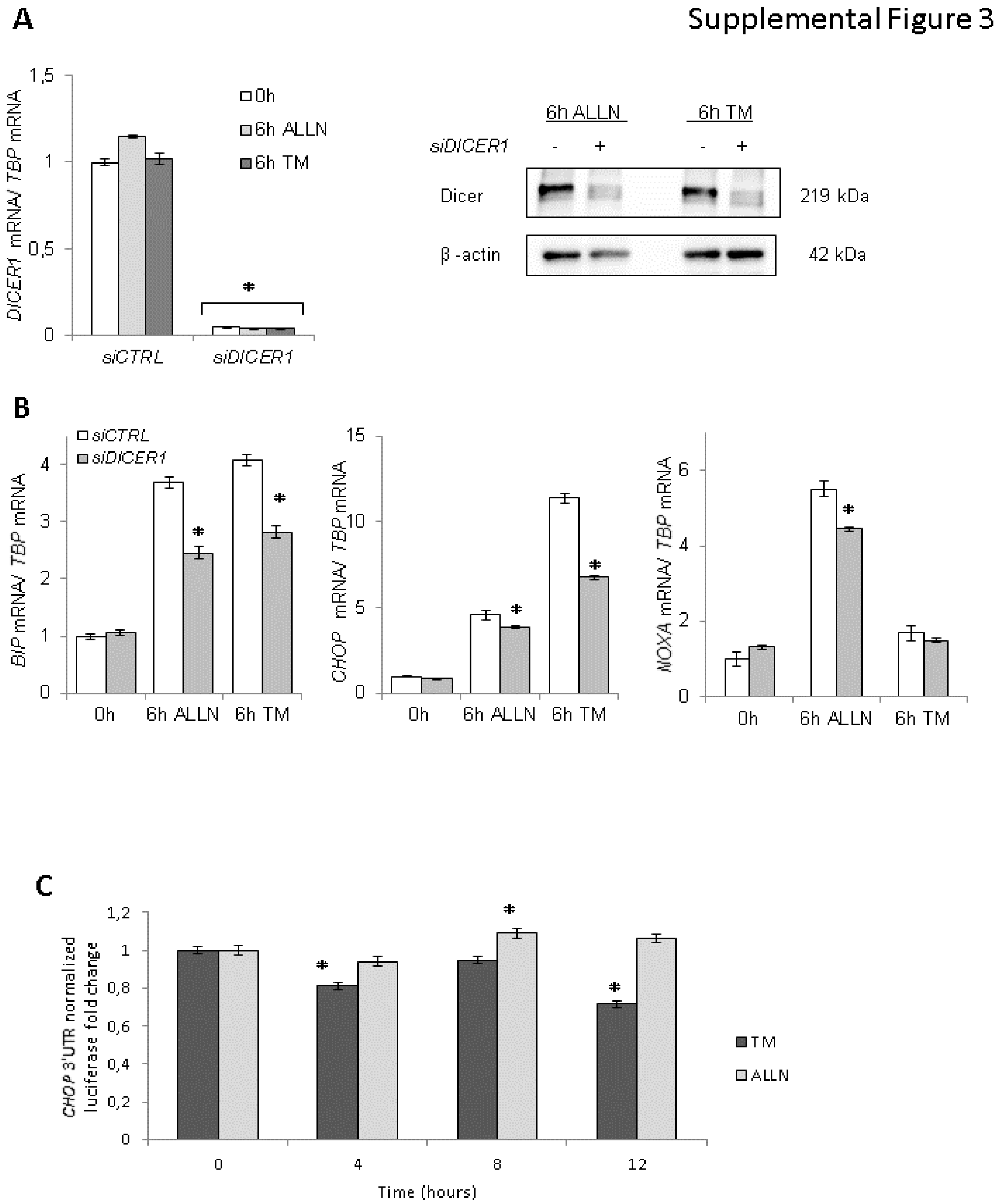

Supplemental Figure 3. (**A**) Efficient *DICER1* expression silencing in 16HBE14o- cells during ER stress. *DICER* mRNA and protein levels were monitored 48 hours post transfection of siRNA against *DICER1* (Silencer Select Pre-Designed siRNA; Gene Symbol: *DICER1*; Cat. No 4390824; siRNA ID:s23756 qPCR: Taqman Assay ID Hs00229023_m1; DICER antibody ab227518, Abcam, https://www.abcam.com/dicer-antibody-ab227518.html). The mRNA levels (n=4) are plotted normalized to *TBP* mRNA levels and expressed as a fold-change over the no stress siRNA control. Error bars represent standard deviations.

Significant changes ($p<0.05$) are marked with an asterisk. (**B**) Silencing of *DICER1* decreases *BIP*, *CHOP*, and *NOXA* mRNA expression levels during ER stress. 16HBEo- cells were transfected with control siRNA or *DICER1* siRNA (and treated with the ER stressors for 6 hours). The mRNA levels (n=4) are plotted normalized to TBP mRNA levels and expressed as a fold-change over the no stress siRNA control. Error bars represent standard deviations. Significant changes ($p<0.05$) are marked with an asterisk. (**C**) *CHOP* mRNA levels are slightly modulated during ER stress through this gene 3'UTR. A human 3′UTR *CHOP* -driven firefly luciferase reporter construct was purchased from GeneCopoeia (HmiT054444; NM_001195053 3'UTR) along with the control vector (CmiT000001-MT06 (miRNA Target clone control vector for pEZX-MT06. This construct contains the human *CHOP* 3′-UTR downstream of firefly luciferase and renilla luciferase as independent control. To test the transcriptional and post-transcriptional activity of the human *CHOP* 3'UTR region, 16HBEo- cells were transfected with the construct described above or with control plasmids provided by GeneCopoeia. Twenty-four hours before the experiments, cells were seeded onto 6-well plates at ~40% confluency and transfected using Lipofectamine 2000 (Invitrogen). Each well received 2 µg of total plasmid DNA and 1 µg of a vector of interest. Following ER stress induction, at the time points indicated, cells were lysed using luciferase assay lysis buffer (Promega) and firefly/*Renilla* luciferase activities were measured using the Dual-Luciferase Reporter Assay (Promega) according to the manufacturer’s protocol. Results in treated cells were plotted as the fold change in arbitrary light units compared with control cells. Experiments were repeated twice (n=16). Significant changes ($p<0.05$) are marked with an asterisk.

***Supplemental Table 3**. Small RNA sequencing of control and ER stress (ALLN and TM treated) samples - samples reads distribution as assigned by Qiagen's Gene Globe Software.*

read set	RA0-170313_S1	RA2-170313_S2	RA6-170313_S4	RA9-170313_S5	RT2-170313_S7	RT6-170313_S9	RT9-170313_S10
total_reads	31 354 275	31 752 920	31 319 258	30 862 543	31 124 652	38 986 167	30 380 389
no_adapter_reads	1 602 063	880 272	664 662	845 421	761 077	835 251	737 210
too_short_reads	16 565 206	18 141 166	18 654 650	17 510 580	18 320 643	26 179 573	18 847 482
UMI_defective_reads	880 921	351 050	262 625	274 559	361 380	331 807	255 865
**miRNA_Reads**	**5 261 627**	**3 464 986**	**2 381 105**	**3 668 855**	**3 495 521**	**2 302 493**	**2 987 764**
hairpin_Reads	4 460	4 053	2 289	3 026	3 307	2 607	2 900
**piRNA_Reads**	**307 907**	**626 829**	**988 076**	**730 945**	**558 259**	**1 162 119**	**516 940**
rRNA_Reads	3 081 914	3 597 696	3 350 358	3 284 418	3 709 229	2 629 466	3 648 879
tRNA_Reads	709 100	1 910 233	3 082 488	2 055 385	1 558 388	3 468 014	1 473 355
mRNA_Reads	309 235	305 872	284 316	239 324	294 655	448 365	276 045
otherRNA_Reads	166 976	178 494	123 716	179 526	138 154	138 623	123 944
notCharacterized_Mappable	989 410	838 046	542 986	786 549	731 950	548 039	582 524
notCharacterized_notMappable	1 476 261	1 454 545	982 196	1 284 359	1 192 456	940 113	927 747

Supplemental Table 4. RNASeq selection of miRNA significantly affected (more than 2-fold) by ER stress that potentially target CHOP and NOXA. *Target prediction was performed with miRDIP software (Tomas Tokar, Chiara Pastrello, Andrea E M Rossos, Mark Abovsky, Anne-Christin Hauschild, Mike Tsay, Richard Lu, Igor Jurisica; mirDIP 4.1—integrative database of human microRNA target predictions, Nucleic Acids Research, Volume 46, Issue D1, 4 January 2018, Pages D360–D370, https://doi.org/10.1093/nar/gkx1144) and only high probability interactions were considered (top 5% of predicted targets).*

	**Fold Regulation (comparing to no stress control)**						Targets *CHOP*	Targets *NOXA*
	**ALLN 2h**	**ALLN 6h**	**ALLN 9h**	**TM 2h**	**TM 6h**	**TM 9h**		
**miRNA ID**	**Fold Regulation**	**Fold Regulation**	**Fold Regulation**	**Fold Regulation**	**Fold Regulation**	**Fold Regulation**		
hsa-miR-101-3p	-4.12	-2.33	-3.26	-1.98	-2.08	-2.41	YES	YES
hsa-miR-106a-5p	-1.76	-1.63	-2.41	-1.68	-1.22	-2.09		YES
hsa-miR-106b-5p	-4.39	-2.56	-6.74	-2.86	-1.68	-3.79		YES
hsa-miR-141-3p	-3.33	-2.05	-3.4	-1.87	-1.79	-2.38		YES
hsa-miR-17-5p	-2.16	-1.94	-2.93	-1.95	-1.36	-2.56		YES
hsa-miR-18a-5p	-2.68	-1.8	-4.59	-1.92	-1.49	-2.21		YES
hsa-miR-19a-3p	-5.77	-3.5	-10.4	-2.92	-3.38	-4.1		YES
hsa-miR-27a-3p	-2.14	-1.94	-3.38	-2.03	-1.48	-2.36		YES
hsa-miR-301a-3p	-4.88	-2.67	-7.33	-2.36	-2.58	-3.23		YES
hsa-miR-31-3p	-2.72	-2.11	-3.94	-1.92	-1.65	-2.56		YES
hsa-miR-324-5p	-1.68	-1.57	-2.54	-1.65	-1.47	-2.24		YES
hsa-miR-542-3p	-3.03	-2	-2.71	-1.55	-1.63	-2.1	YES	

Supplemental Figure 4. Uncropped gels and blots for Figure 3A and Figure 4B, in order of appearance.

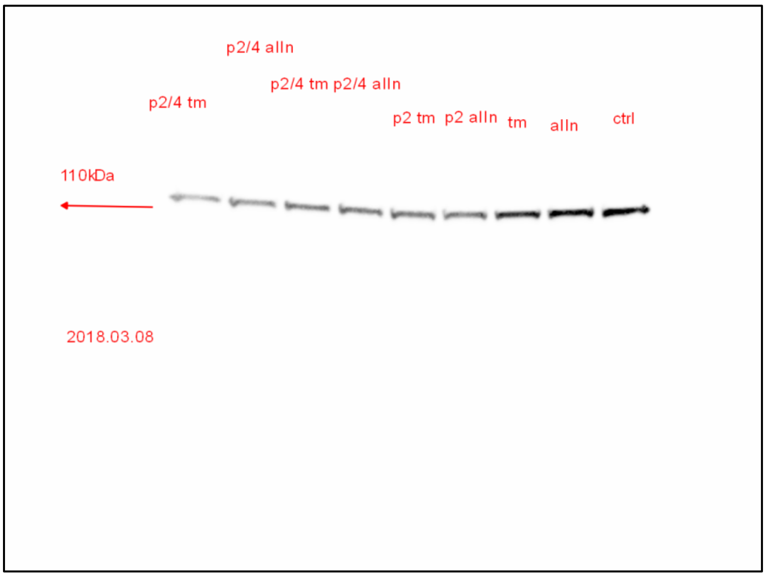

Figure 3A. The uncropped blot for PIWL2 Exposition in seconds 0.124 (Auto - Intense Bands).

Figure 3A.The uncropped stain free gel; m- Kaleidoscope marker (Bio-Rad)

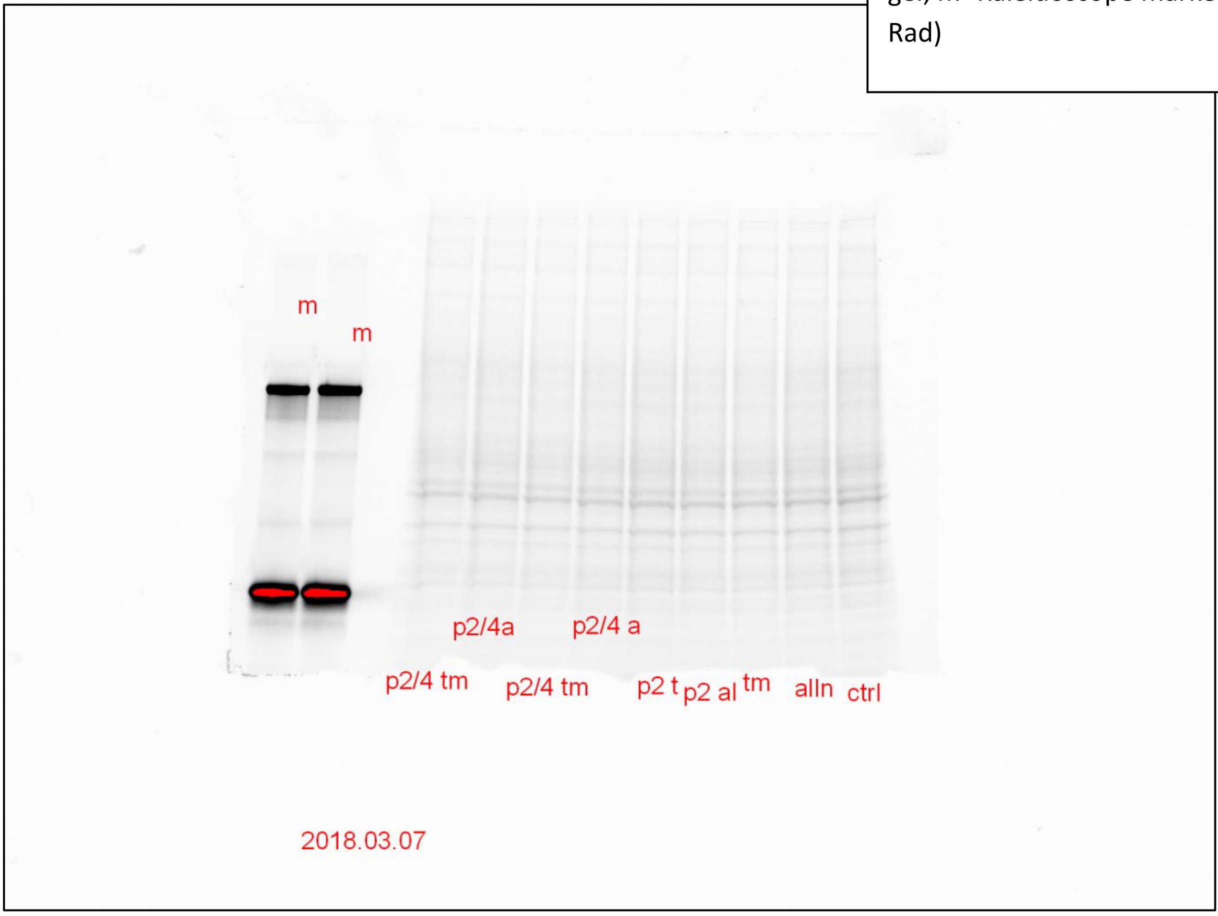

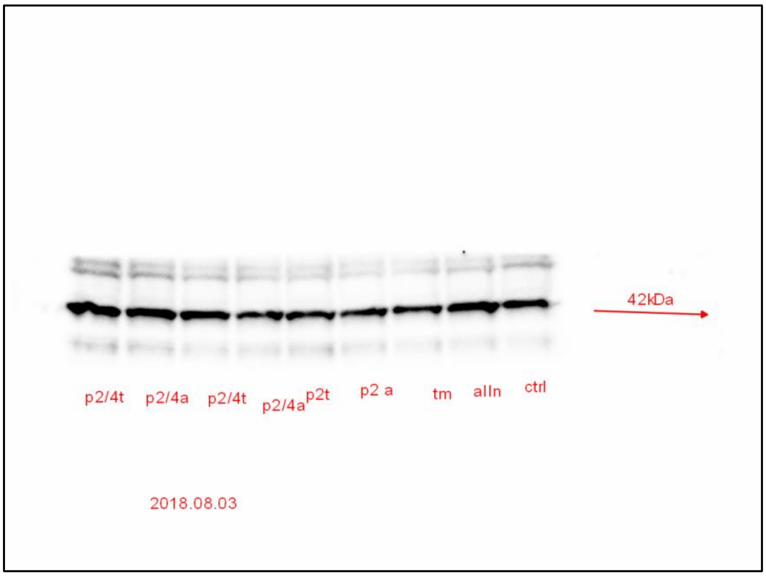

Figure 3A. The uncropped blot for beta Actin for PIWIL2 experiment; Exposition in seconds: 5.738 (Auto - Intense Bands).

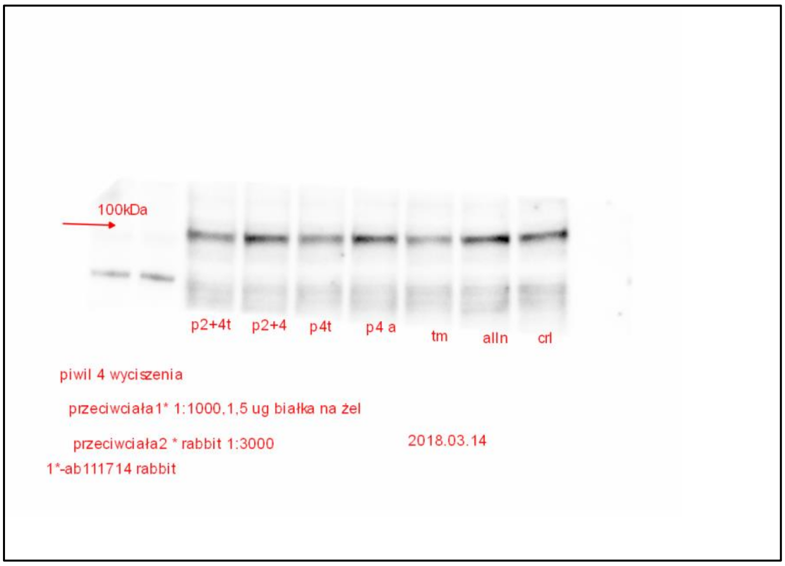

Figure 3A. The uncropped blot for PIWL4 Exposition in seconds 3.262 (Auto - Intense Bands).

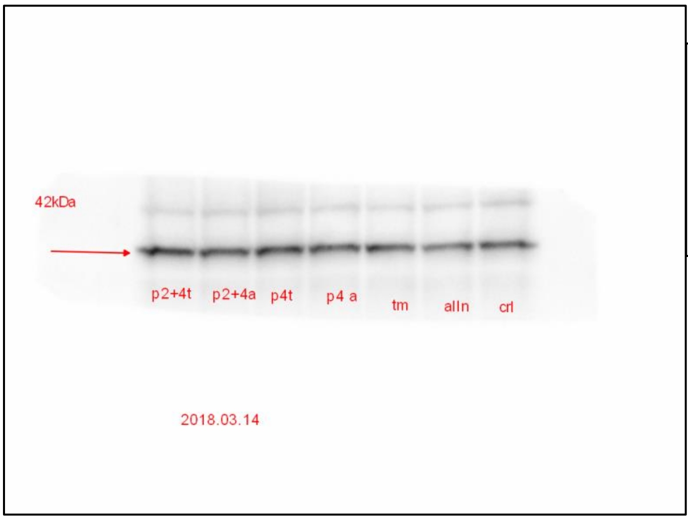

Figure 3A. The uncropped blot for beta Actin for PIWIL4 experiment; Exposition in seconds: 5.114 (Auto - Intense Bands).

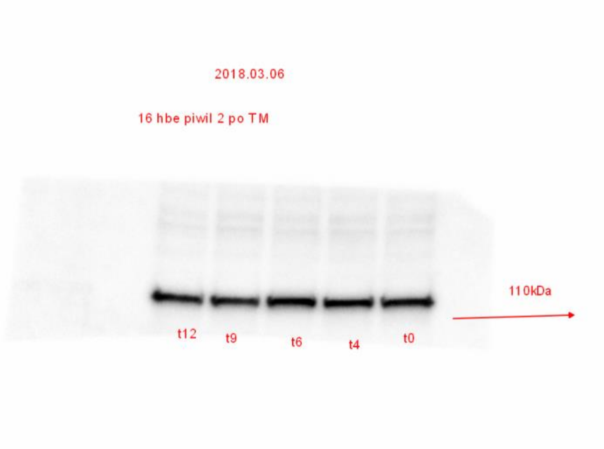

Figure 4B. The uncropped blot for PIWL2 TM Exposition in seconds 0.351 (Auto - Intense Bands)

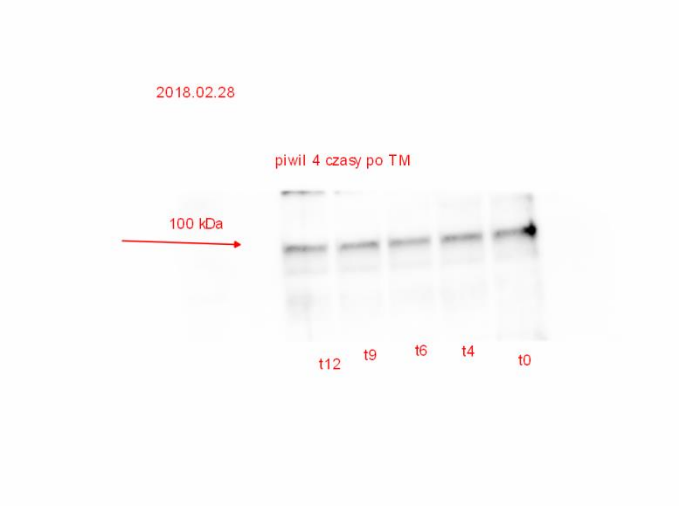

Figure 4B. The uncropped blot for PIWL4 TM Exposition in seconds 1.750 (Auto - Intense Bands)

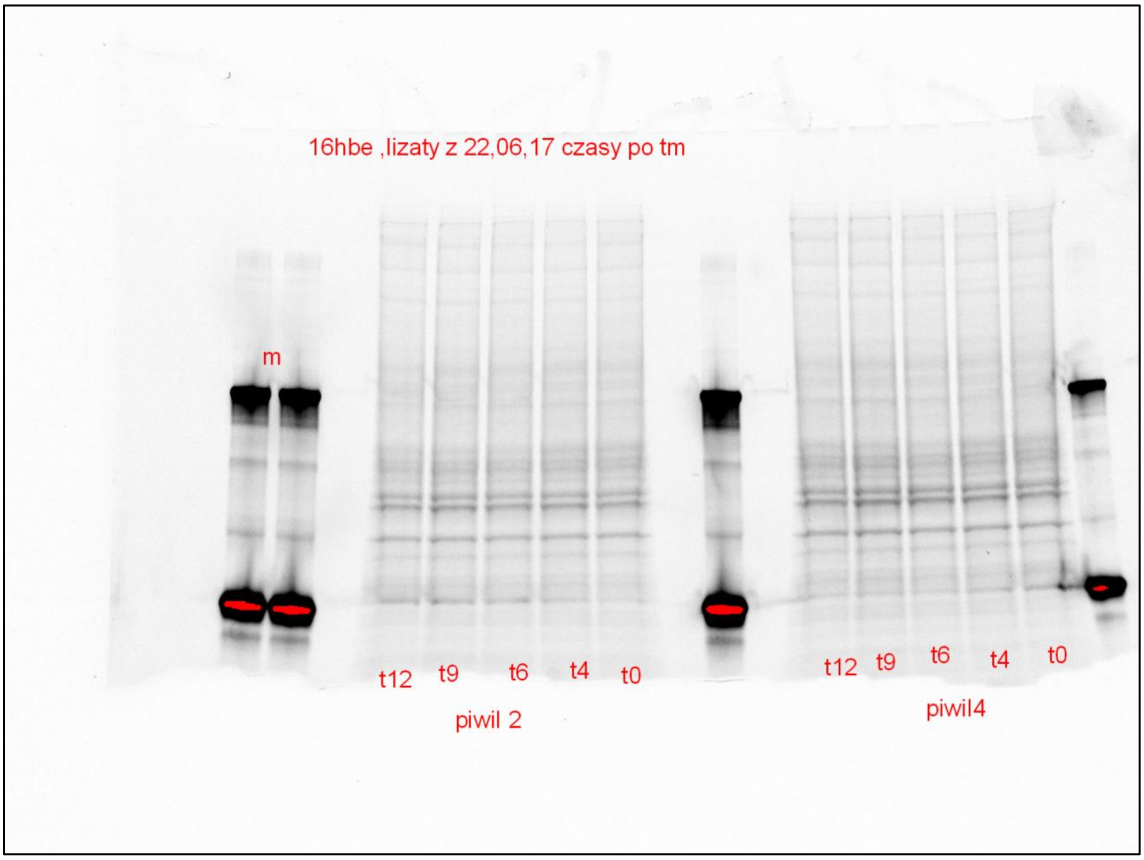

Figure 4B. The uncropped stain free gel; m- kaleidoscope marker (Bio-Rad)

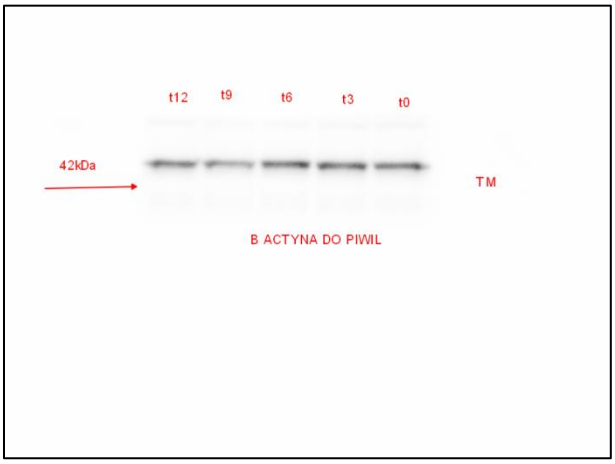

Figure 4B. The uncropped blot for beta Actin for PIWIL TM experiment; Exposition in seconds: 3.425 (Auto - Intense Bands).

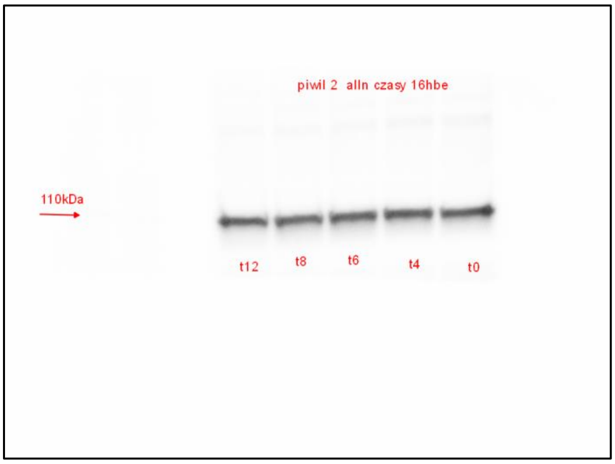

Figure 4 B. Uncropped blot for PIWL2 ALLN Exposition in seconds 0.203 (Auto - Intense Bands)

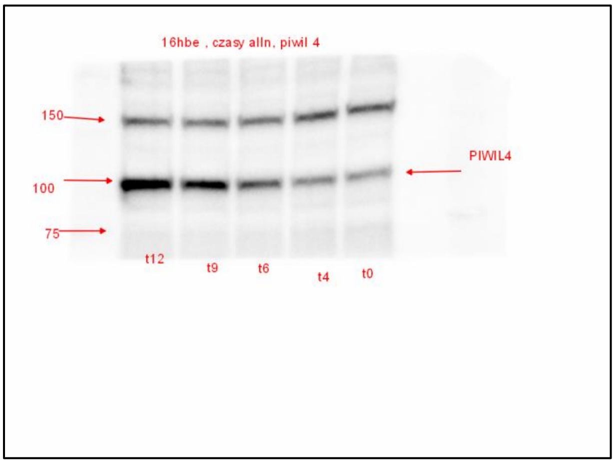

Figure 4 B. Uncropped blot for PIWL4 ALLN Exposition in seconds 0.524 (Auto - Intense Bands)

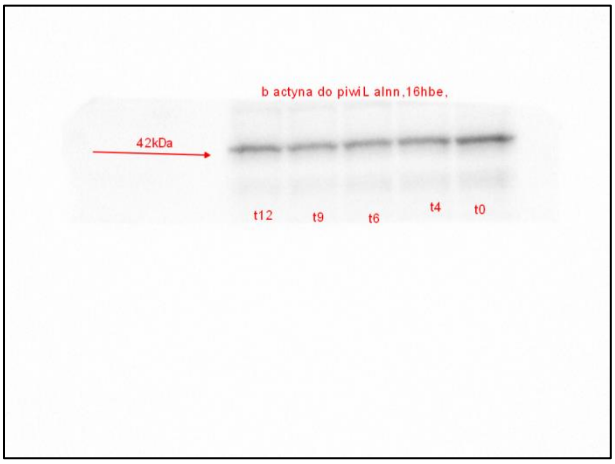

Figure 4B. Uncropped blot for beta Actin for PIWIL ALLN experiment; Exposition in seconds: 1 (Auto - Intense Bands).

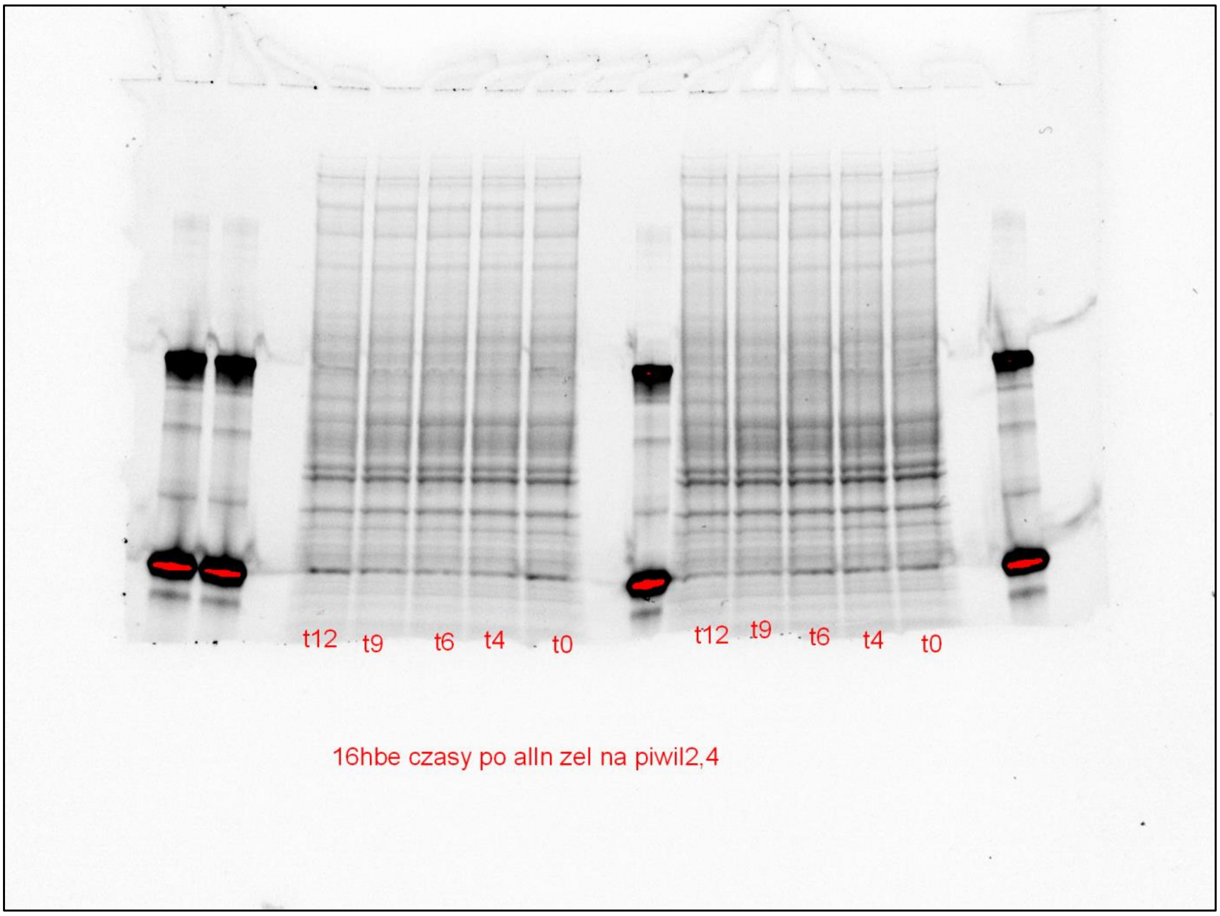

Figure 4B. Uncropped stain free gel

m- Kaleidoscope marker (Bio-Rad)

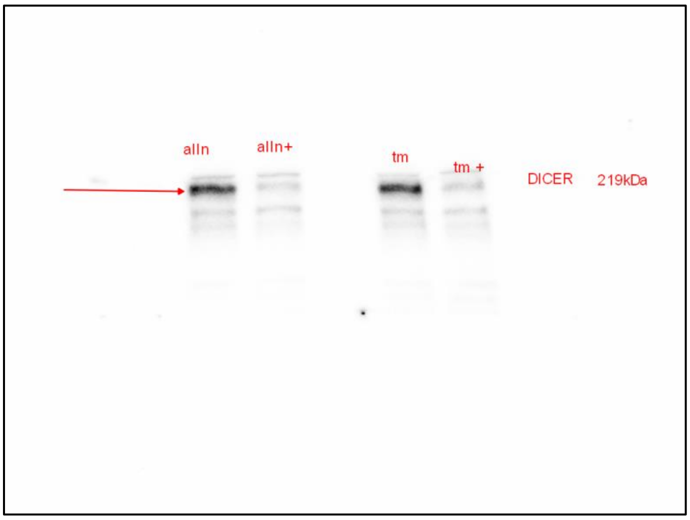

Figure S3. Uncropped blot for DICER Exposition in seconds 63,971 (Auto - Intense Bands)

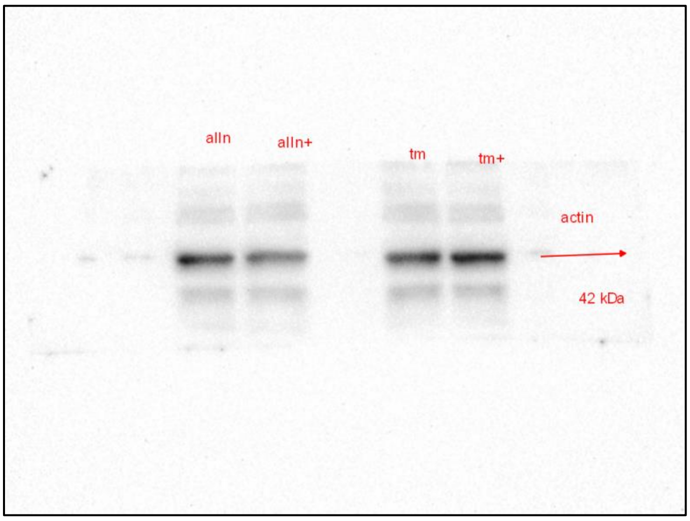

Figure S3. Uncropped blot for Actin Exposition in seconds 63,971 (Auto - Intense Bands)

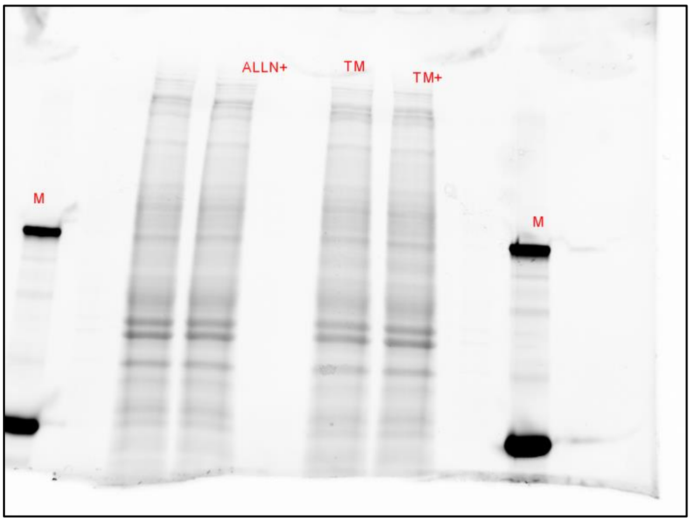

Figure S3. The uncropped stain free gel

m- Kaleidoscope marker (Bio-Rad)
